# Parliament and the Profession

**Published:** 1915-11-20

**Authors:** 


### PARLIAMENT AND THE PROFESSION.

### The Nurse's Wardrobe.

Is the military hospital nurse able to buy her uniform on the best possible terms? This was the gist of a question asked in the House of Commons on November 11 by Mr. Yeo, who suggested that nurses were compelled to buy their uniforms and necessaries from West-End firms who have to pay heavy rents—cloth from one shop, millinery from another, linen from another, and so on. Mr. Tennant replied that the nurses attached to Queen Alexandra's Imperial Military Nursing scheme did not purchase their uniform piecemeal. One firm in London stocked all they required, and a similar arrangement had been made in some of the large provincial towns.

### An Alleged Wastage of Medical Men.

In the House of Commons on November 11 Mr. Pringle suggested that a large number of Army doctors are engaged in clerical work, but Mr. Tennant was doubtful whether this was so. The point arose out of a suggestion made by Mr. James Mason that in view of the shortage of medical officers for the Army it would be advisable to transfer the greater part of the administrative work of the Army Medical Department and the R.A.M.C. to laymen, and to appoint laymen for administrative purposes to the commissioned ranks of the R.A.M.C.

### Recovery of Fees from Doctors.

Under instructions given in December last fees are recovered from doctors who have passed men into the Army as medically fit who have subsequently been discharged as medically unfit. This was the effect of a statement made in the House of Commons on November 10 by Mr. Forster in reply to a question by Mr. Hogge.

### Promotion of Army Doctors

In the House of Commons on November 10, Sir Daniel Goddard asked whether the decision to promote to the rank of captain all lieutenants of the Territorial Force, R.A.M.C., who had given six months' mobilised service, with effect from April 1 last, was being carried out. Mr. Forster replied that promotions are carried out immediately on receipt of individual recommendations; the majority of these have already been put forward and have been duly gazetted.

### Diseases in the Army.

In the House of Commons on November 9, Mr. Will Thorne asked the Under-Secretary of State for War if he would state the number of cases of and deaths from pyrexia, trench fever, dysentery, typhus fever, pneumonia, cerebrospinal meningitis, typhoid fever, and paratyphoid fever in the British Army since August 1, 1914. Mr. Tennant replied that this information was not available.

### The Royal Army Medical Corps.

The total number of Army medical officers is now 9,626. The present number of Territorial Force medical officers which is included in the above total is 2,474. A statement to the above effect was made in the House of Commons by the Under-Secretary of State for War on November 11.

### The Treatment of Insured Consumptives.

A series of questions alleging deficits in the Insurance Act funds has again been asked in the House of Commons. To the majority of these it is unnecessary to allude, and it will suffice to quote the statement made by Mr. Charles Roberts on November 11, that the funds available for the treatment of insured persons suffering from tuberculosis are provided by statute, and "there is no foundation whatever for the misleading statement, to which publicity has recently been given, that the provision of these funds is in danger of cessation."

### Maternal Mortality.

A somewhat curious question has been asked in the House of Commons by Sir J. D. Rees. It suggested that notwithstanding the grant of maternity benefit under the Insurance Act maternal mortality had increased since the scheme came into force. If this were so, Sir J. D. Rees desired to know whether this benefit was to be continued "after it has been proved by experience that pecuniary aid does not result in the anticipated improvement." Mr. C. Roberts, in his reply, said he was not in possession of any information which established the suggestion that maternal mortality had increased, nor was sufficient experience available to support the conclusion that no improvement had been experienced since the Act came into force.

